# Fieldwork for public health responses during pandemics: lessons from the New South Wales Health experience with COVID-19

**Published:** 2022-04-08

**Authors:** Laksmi S. Govindasamy, Anthony Zheng, Ming Chen, Debbie Chia, Paola Garcia, Chaturangi Yapa, Tara Smith

**Affiliations:** a New South Wales Ministry of Health, New South Wales, Australia.; b Australian National University, Canberra, Australia.

## Abstract

**Problem:**

Fieldwork is a vital component of public health emergency response, yet little has been published on undertaking fieldwork safely. Safety is of particular importance with emerging pandemic viruses, which can pose additional risks to public health fieldwork staff.

**Context:**

During a pandemic, surge health staff may be drawn from diverse professional backgrounds; they may have limited experience in fieldwork or be unfamiliar with the risks posed by a novel virus. Novel pathogens pose dangers to fieldwork staff, particularly when there are global or local shortages of personal protective equipment.

**Action:**

During the coronavirus disease 2019 (COVID-19) pandemic, New South Wales (NSW) Health’s Public Health Emergency Operations Centre (PHEOC) deployed staff for fieldwork in a range of settings. The PHEOC developed a protocol to systematize planning, risk assessment and management for COVID-19 fieldwork. The protocol was accompanied by training, discussion exercises and debriefs to support PHEOC fieldwork staff.

**Lessons learned:**

Effective fieldwork is an essential component of outbreak investigation and management, including stakeholder management. Here, we share and discuss key elements of the NSW Health protocol to support fieldwork during outbreak responses for emerging communicable diseases across various resource contexts. Limited understanding of novel viruses, particularly in the early phases of a pandemic, must be considered in decisions to deploy fieldwork staff and implement precautionary risk mitigation approaches. Planning is essential to protect staff and ensure ethical allocation of resources. Through appropriate selection of teams and training, surge staff can be supported to effectively conduct fieldwork.

## Problem

Public health responses to outbreaks can involve fieldwork to support outbreak investigation and implementation of control measures (where “fieldwork” means deploying staff to outbreak sites to support the public health response). Fieldwork is common in public health, yet little has been published on how to safely conduct it. The emergence and spread of severe acute respiratory syndrome coronavirus 2 (SARS-CoV-2), the virus responsible for coronavirus disease 2019 (COVID-19), highlights the challenges posed by novel viruses for public health authorities coordinating fieldwork. Public health practitioners are a vital but scarce resource during pandemic response. Beyond workplace obligations, an effective public health response must assess, mitigate and control occupational exposure of fieldwork staff. Failure to achieve this can place staff and the community at risk of infection, with potentially serious consequences.

Fieldwork can be particularly difficult when responding to a condition for which there is an evolving knowledge base. Our understanding of SARS-CoV-2 transmission has changed rapidly over the course of the pandemic (e.g. changing definitions for cases and close contacts of COVID-19 in Australia’s national guidelines). (*1*) The possibility of asymptomatic and pre-symptomatic transmission and the broad range of clinical symptoms (*2*) can lead to retrospective recognition of infectious exposures during fieldwork, given that new cases may be identified after symptoms develop or following broader testing.

Here, we present learnings from the development of the New South Wales (NSW) Health protocol: *Preparing for COVID-19 fieldwork: protocol for PHEOC staff* (hereafter, the protocol) and share its framework and checklists to support risk management for other jurisdictions. The protocol was developed for COVID-19 fieldwork in NSW, but we believe this approach can be readily adapted for application to other infectious diseases and resource contexts.

## Context

Responsibility for coordinating fieldwork to support public health investigations and responses during the COVID-19 pandemic lay with the NSW Ministry of Health’s Public Health Emergency Operations Centre (PHEOC) and the public health units (PHUs) of local health districts. At NSW Health, public health fieldwork is typically undertaken by PHUs. Fieldwork supports investigations of infectious disease outbreaks and environmental exposures, implementation of public health management and coordination of response management. However, in the context of a novel virus, there were specific challenges:

Pandemic responses often involve surge staff, who may have limited public health or fieldwork experience. (*3*)
Experienced public health staff may be unfamiliar with the risks posed by a new virus and the specific infection prevention and control (IPC) procedures required, such as personal protective equipment (PPE). (*4*) Although PPE training is routinely provided as part of orientation for new employees in Australian health systems, less than half of surveyed providers report annual or regular refresher training. (*5*) For non-clinical public health staff, there may be even fewer opportunities for routine IPC training.
The pandemic context typically requires urgent decision-making and rapid responses, potentially against a background of an overwhelmed health system and resource scarcity that presents ethical challenges. (*6*)

## Action

During the NSW Health COVID-19 pandemic response, the PHEOC’s operations team undertook fieldwork in various settings, including residential aged-care facilities, schools, cruise ships, workplaces, airports and hotels. Each setting required relationship building through consultation with different stakeholders. Fieldwork activities included site assessment to consider potential transmission dynamics and to inform contact tracing; screening to identify individuals at risk of COVID-19; collection of samples for SARS-CoV-2 testing; and IPC assessments. Often, fieldwork staff were also required to provide immediate public health advice, support outbreak management decisions and communicate with stakeholders (both onsite and offsite). Decisions to deploy staff for fieldwork were generally required urgently, typically within 1–12 hours.

**Fig. 1:**
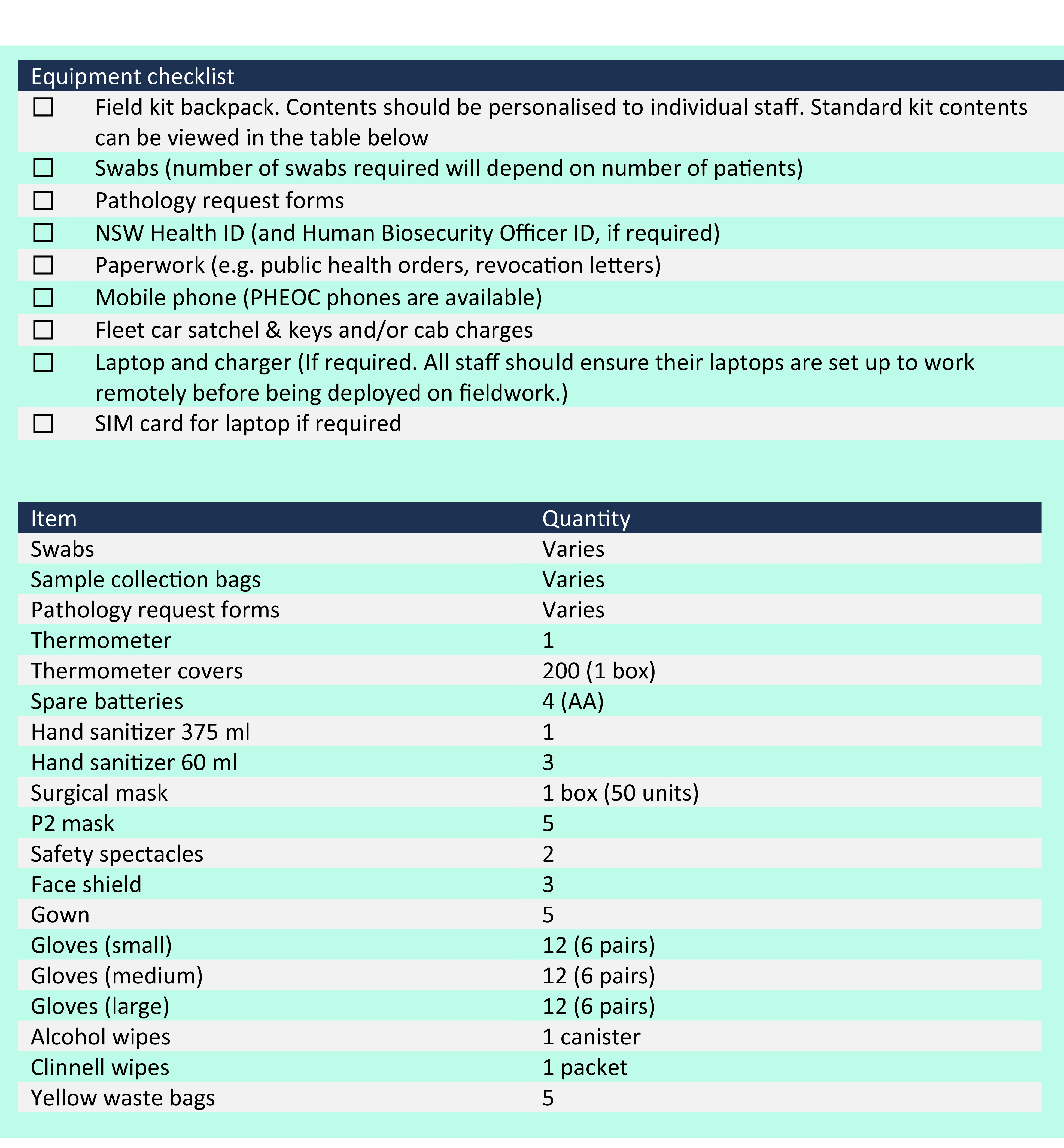
Equipment checklist and standard kit contents, NSW Health

The PHEOC iteratively developed the protocol to guide and support fieldwork teams as understanding of SARS-CoV-2 evolved. For example, when the protocol was first drafted, it was not clear whether asymptomatic transmission was possible or was a key driver of transmission. Once such transmission was recognized, we strengthened the protocol’s approach to IPC accordingly. Issues identified from fieldwork deployment debriefs were used (with reference to the NSW Health risk management policy) (*7*) to develop and test the protocol with fieldwork staff, to ensure that solutions were pragmatic and realistic. Thus, the protocol was repeatedly piloted, tested and revised, and drew on the lived experiences of deployed staff. Additionally, the PHEOC operations team facilitated fieldwork training for surge staff, including tabletop discussion exercises, after which the protocol and field kits stocked for deployments were further improved and incorporated into an equipment checklist (**Fig. 1**).

Incorporating IPC measures was identified as a vital component of risk mitigation, along with the recognition of insufficient capacity within the PHEOC to provide IPC training. IPC experts and clinical nurse consultants from the NSW Government’s Clinical Excellence Commission (CEC) were engaged to provide IPC training and assessment to surge staff. The CEC produced guidelines for IPC in clinical settings in NSW Health, (*4*) and their staff directly supported PHEOC’s public health fieldwork activities, including onsite IPC assessments. The CEC delivered tailored IPC training, including certification for fieldwork staff in safe use of PPE requirements for COVID-19 (standard, contact, droplet and aerosol precautions) (*8*) and practical guidance on identifying and managing breaches of PPE, and the establishment of field donning and doffing stations.

[**insert** Figure 1]

**Fig. 2:**
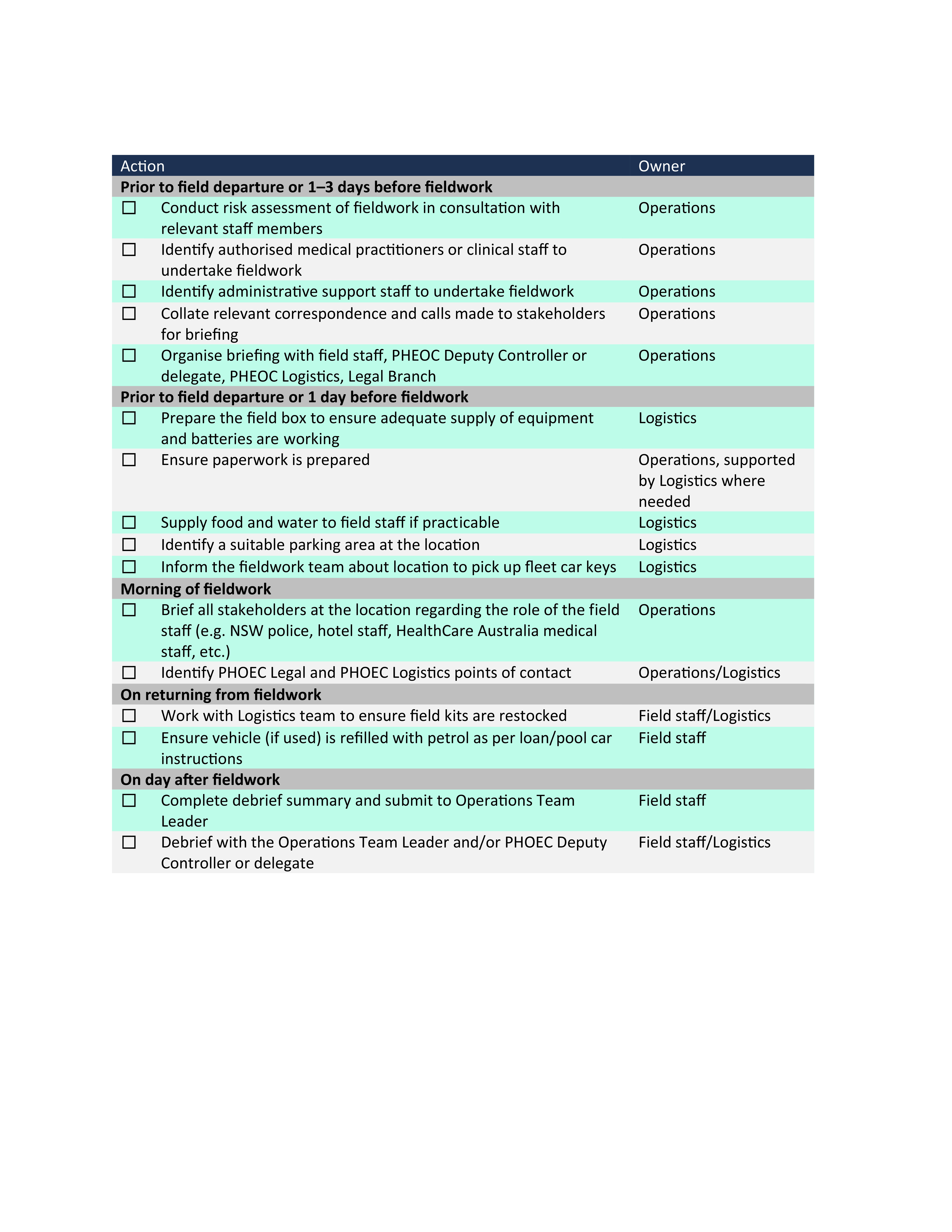
Checklist of key actions to support fieldwork deployment, NSW Health

**Fig. 3:**
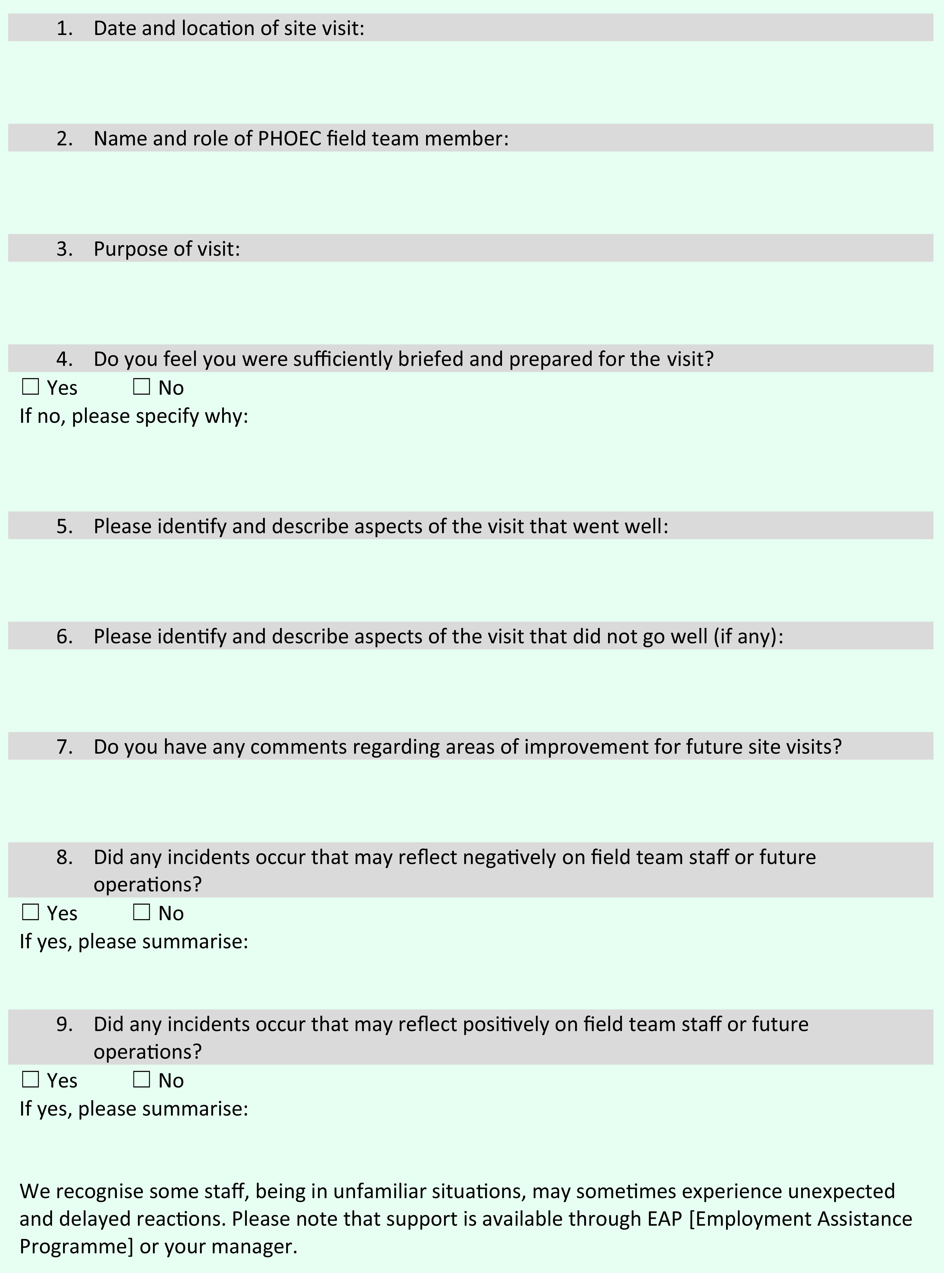
Post-fieldwork debrief form, NSW Health

Developing the protocol highlighted the importance of preparation, risk assessment and team briefings before fieldwork activities, systematized by the development of an action checklist (**Fig. 2**). A key question posed during the risk assessment process was whether there were any effective alternatives to undertaking fieldwork that would minimize risks to staff (e.g. videoconferencing, using resources such as maps of facilities or using a risk assessment tool). Likewise, the opportunities for other staff to learn from the experiences of fieldwork staff and to make further improvements to the protocol were formalized through the development of a structured debrief document (**Fig. 3**).

[**insert** Figure 2]

[**insert** Figure 3]

## Lessons learned

In our experience, effective fieldwork can confer benefits beyond the investigative information gathered in the field to guide outbreak response efforts. Importantly, fieldwork plays a crucial role in establishing cooperative relationships with staff at facilities and organizations, which are integral components of successful outbreak management. For example, some residential aged-care facilities experienced a significant increase in workload during outbreaks, with reduced staff, increased IPC requirements, increased reporting demands and media attention. In such cases, fieldwork staff were able to develop collaborative relationships that were especially important during prolonged outbreak responses. This was in addition to supporting the investigation of outbreak sources; training, assessment and monitoring of IPC measures; and identification of further cases through collection of SARS-CoV-2 samples and review of clinical records. Onsite deployment of public health fieldwork staff can support residential aged-care facility staff and can expedite outbreak investigation and implementation of effective control measures.

Fieldwork can pose challenges for the safety of public health staff. During the COVID-19 pandemic in NSW, identification of potential exposures to SARS-CoV-2 required fieldwork staff to self-isolate as close contacts, as per national guidelines. (*1*) A risk assessment determined that the likelihood of exposure was relatively low; however, the potential consequences – specifically, further transmission to colleagues in the PHEOC – were considered severe. This experience catalysed the prioritization of systematic preparation to reduce the risk of such exposures. Anecdotally, a revised protocol that strengthened systematic preparation (e.g. by including checklists of key PPE required) helped to make fieldwork staff feel safer during their deployments.

We recommend the routine inclusion of briefings before and after fieldwork as a way to support fieldwork staff. Our recommended approach is for a senior public health practitioner to be dedicated to supporting the field team and facilitating briefings and debriefs. A briefing is an opportunity to convene the fieldwork and support teams; clarify roles, objectives and responsibilities; and confirm risk assessment and mitigation strategies. Clear lines of communication were established for fieldwork staff to escalate concerns and obtain decision-making support during deployment. In facilitating the debrief, it is important to create space for open disclosures of challenges encountered, so that learnings from each field visit can be incorporated into institutional knowledge. Before the development of the protocol, debrief sessions were (perhaps appropriately) a low priority in the context of an ongoing emergency response. However, after implementing the protocol, debrief sessions became routine and normalized, which facilitated further improvements to staff experience, protocol development, and the reporting of adverse experiences and events.

In our view, maintaining the safety of team members must be the primary priority during fieldwork. IPC, onsite risk assessments and requisite modification of activities should be undertaken immediately before and during the field visit to reduce the risk of exposures. PPE should be considered a last line of defence, as a mental prompt for fieldwork staff to first consider other measures to reduce risk (e.g. videoconferencing, using tools such as maps of facilities or risk management matrices, physical distancing and referring sample collection to experienced clinical staff). Where there is a need for PPE, fieldwork staff require comprehensive training in the use of PPE well in advance of a proposed field visit. This training should incorporate best practice techniques and pragmatic approaches (e.g. recognizing and appropriately managing PPE breaches).

Public health decisions for COVID-19 generated highly emotive community responses and close media interest, particularly where isolation or travel restrictions were required. We therefore recommend deploying fieldwork staff in teams of at least two for safety and security reasons. Pairs of staff can also support identification of PPE breaches and supervise donning and doffing of PPE for each other. We suggest that teams include senior public health practitioners where possible. Senior staff can provide important training and support for junior or surge staff, and their experience can facilitate attainment of fieldwork goals. Although multidisciplinary public health practitioners can effectively undertake fieldwork, we recommend inclusion of at least one clinical team member in case of unexpected requirements for clinical sampling of potential cases or other clinical needs. Factors to consider when composing the team include individual scope of practice of team members, and professional and organizational liability for public health decisions, including implementing public health orders and providing clinical care in the field setting.

In an ideal setting, there would be physical separation of fieldwork staff from other staff in the workplace, to minimize risk of workplace transmission. However, in practice, the scarcity of public health personnel may preclude ongoing separation. Nonetheless, remote work and rostering arrangements could be optimized to reduce the exposure of other public health staff during the incubation period immediately following potential exposure from fieldwork. Where this cannot be achieved, it becomes imperative for fieldwork staff to be supported to immediately openly disclose any potential exposures incurred during fieldwork or the evolution of subsequent symptoms. To ensure there are no incentives to avoid open disclosure, we recommend establishing organizational processes, such as access to appropriate paid leave arrangements and support to manage self-isolation. For example, NSW Health, alongside other Australian jurisdictions, has made alternative accommodation options available to support health-care workers who are required to self-isolate, to reduce the risk of household transmission. (*9*, *10*)

## Discussion

There are currently no publications exploring the benefits and challenges of public health fieldwork and optimal processes for such fieldwork. The NSW Health experience highlighted that fieldwork is a crucial component of effective public health responses during the COVID-19 pandemic; we are therefore sharing our learnings from fieldwork in pandemic settings. We believe the principles and approach described in the protocol can be readily adapted, implemented and scaled to support fieldwork in response to other outbreaks, including future emerging infectious diseases. These approaches may also be applicable across a wide range of resource and health system contexts.

We recognize that in low-resource contexts, safely conducting fieldwork can be especially challenging but it remains crucial, particularly to prevent transmission among limited staff. An important risk mitigation strategy is implementation of IPC measures, including appropriate use of recommended PPE. The global impacts of the COVID-19 pandemic have caused significant shortages of PPE, requiring rational use and prioritization. (*11*) However, training in PPE by IPC experts can support judicious and effective use of limited supplies. Where possible, we recommend the deployment of senior public health staff whose experience and authority can contribute to effective fieldwork. However, such experienced staff are themselves limited resources in a pandemic context, and ensuring their safety and capacity to continue contributing to the broader pandemic response is essential. It is imperative that health authorities ensure effective risk management of fieldwork, and that fieldwork is considered a part of workplace obligations. We encourage public health practitioners in low-resource contexts to consider the principles discussed in this paper when developing context-specific fieldwork protocols.

It is also important to acknowledge the potential impact on staff well-being following fieldwork and especially following occupational exposure. In the early period of the pandemic response, the complications and risks of COVID-19 were poorly understood, causing substantial anxiety among health-care workers, particularly regarding the risk of transmission to their families. (*12*) Fieldwork may itself involve challenging stakeholder management and other stressors, after which staff may experience a delayed psychological response; hence, they should be supported to obtain formal assistance as required. Before fieldwork deployment, managers should consider whether staff have pre-existing medical conditions that may make them more vulnerable to severe illness and, if so, make provisions to mitigate risks.

## Conclusion

Deploying public health staff to conduct fieldwork in various outbreak settings is an important aspect of the NSW public health response during the COVID-19 pandemic. Our experience and lessons learned in developing a protocol for effectively equipping staff with the skills, knowledge and expertise to perform fieldwork safely may assist other jurisdictions in their public health response and control efforts, both with COVID-19 and other outbreaks. To receive a copy of the protocol, please contact the corresponding author.
